# Supplementary material for: Reversible hydrogen spillover in Ru-WO3-x enhances hydrogen evolution activity in neutral pH water splitting
Source: Nat Commun. 2022 Sep 14;13:5382. doi: 10.1038/s41467-022-33007-3 (PMC9474501; doi:10.1038/s41467-022-33007-3)
Supplement: Supplementary file 1 — Supplementary Information [file 41467_2022_33007_MOESM1_ESM.pdf]

Supplementary Information

Reversible hydrogen spillover in Ru-WO_{3-x} enhances hydrogen evolution activity in neutral pH water splitting

Jiadong Chen[1,2,†], Chunhong Chen[1,†], Minkai Qin [1,†], Ben Li[1], Binbin Lin[1], Qing Mao[3], Hongbin Yang[2], Bin Liu[2,4,*] and Yong Wang[1,*]

[1]Advanced Materials and Catalysis Group, Center of Chemistry for Frontier Technologies, State Key Laboratory of Clean Energy Utilization, Institute of Catalysis, Department of Chemistry, Zhejiang University, Hangzhou 310028, P. R. China

[2]School of Chemical and Biomedical Engineering, Nanyang Technological University, Singapore 637459, Singapore.

[3]School of Chemical Engineering, Dalian University of Technology, Dalian 116024, Liaoning, P. R. China.

[4]Division of Chemistry and Biological Chemistry, School of Physical and Mathematical Sciences, Nanyang Technological University, Singapore 637371, Singapore.

[†]These authors contributed equally.

Email: liubin@ntu.edu.sg (B. Liu) and chemwy@zju.edu.cn (Y. Wang)

This file includes:
Supplementary Notes 1 to 2
Supplementary Tables 1 to 9
Supplementary Figures 1 to 32
Supplementary References 1 to 26

Table of Contents

1. Supplementary Notes…………………..……………………………………….... 3
2. Supplementary Tables…...…………………………………….……………...….. 5
3. Supplementary Figures.…………………………………………………...…… 6
4. Supplementary References……………..…………….........……….………… 35

Supplementary Notes

Supplementary Note 1. Microkinetic modelling of HER on Ru/C and Ru-WO_{3-x}.

In alkaline/neutral environment, Markovic et al. proposed that H_2O rather than H^+ is the reactant of HER, which directly dissociates on the surface of HER catalyst[1]. However, not all water molecules at the catalyst/electrolyte interface are able to participate in HER. It was suggested that the noncovalent interactions between the oxophilic sites on the surface of catalyst and the water molecules could promote the activity of HER. Furthermore, Wang et al. found that the hydrated alkali cations could adsorb in the catalyst's double layer, which *in-situ* activated the H-OH bond through the hydration effect, promoting dissociation of water molecules[2]. Therefore, it is deduced that water molecules need to be activated ($H_2O_{act,ads}$) before they can participate in the HER in alkaline/neutral environment.

With consideration of H_2O activation, a microkinetic model for HER in neutral environment was constructed as shown in Supplementary Table 1. The kinetics parameters were obtained by fitting the model with steady-state internal resistance (IR) corrected polarization curves using a global optimization method, which composes of a random search using genetic algorithm and a local optimization employing trust-region-reflective algorithm. All simulations were performed using a commercial software package (MATLAB R2010b, the MathWorks Inc.). The relative error tolerance for the initial value problem of ordinary differential equations was set as 10^{-6}.

Supplementary Tables 1 and 2 list the elementary reaction steps and the enrolled reaction rate equations of HER in neutral electrolyte, where r is the elementary reaction rate [$mol \cdot m^{-2} \cdot s^{-1}$], K is the reaction rate constant [$mol \cdot m^{-2} \cdot s^{-1}$], c is the concentration [$mol \cdot m^{-3}$], θ is the coverage, α is the symmetry coefficient, η is the overpotential [V], R is the gas constant [$J \cdot mol^{-1} \cdot K^{-1}$], T is the temperature [K], θ subscripts $H_2O_{act,ads}$ and H_{ads} represent the activated water molecule and adsorbed H intermediate, respectively.

Supplementary Table 1. Elementary reaction steps of HER in neutral medium.

$M+H_2O \underset{K_{102}}{\overset{K_{101}}{\rightleftharpoons}} M\cdot H_2O_{act,ads}$	(1)
$M\cdot H_2O_{act,ads}+e^- \underset{K_{202}}{\overset{K_{201}}{\rightleftharpoons}} M\cdot H_{ads}+OH^-$	(2)
$M\cdot H_{ads}+H_2O+e^- \underset{K_{302}}{\overset{K_{301}}{\rightleftharpoons}} M+H_2+OH^-$	(3)
$r_1=K_{101}\cdot\left(1-\theta_{H_2O_{act,ads}}-\theta_{H_{ads}}\right)$	(4)
$r_{-1}=K_{102}\cdot\theta_{H_2O_{act,ads}}$	(5)
$r_2=K_{201}\cdot\theta_{H_2O_{act,ads}}\exp\left(\frac{\alpha F\eta}{RT}\right)$	(6)
$r_{-2}=K_{202}\cdot\theta_{H_{ads}}\cdot\left(\frac{c_{OH^-}}{c^{\Theta}}\right)\cdot\exp\left(-\frac{(1-\alpha)F\eta}{RT}\right)$	(7)
$r_3=K_{301}\cdot\theta_{H_{ads}}\cdot\exp\left(\frac{\alpha F\eta}{RT}\right)$	(8)
$r_{-3}=K_{302}\cdot\left(1-\theta_{H_2O_{act,ads}}-\theta_{H_{ads}}\right)\cdot\left(\frac{c_{OH^-}}{c^{\Theta}}\right)\exp\left(-\frac{(1-\alpha)F\eta}{RT}\right)$	(9)

Supplementary Table 2. Reaction rate equations for HER in neutral medium.

$C_{H_2O_{act,ads}}\frac{d\theta_{H_2O_{act,ads}}}{dt}=r_1-r_{-1}-r_2+r_{-2}$	(10)
$C_{H_{ads}}\frac{d\theta_{H_{ads}}}{dt}=r_2-r_{-2}-r_3+r_{-3}$	(11)
$C_{dl}\frac{dE}{dt}=j(t)-\left[(r_2-r_{-2})+(r_3-r_{-3})\right]\cdot F/n$	(12)

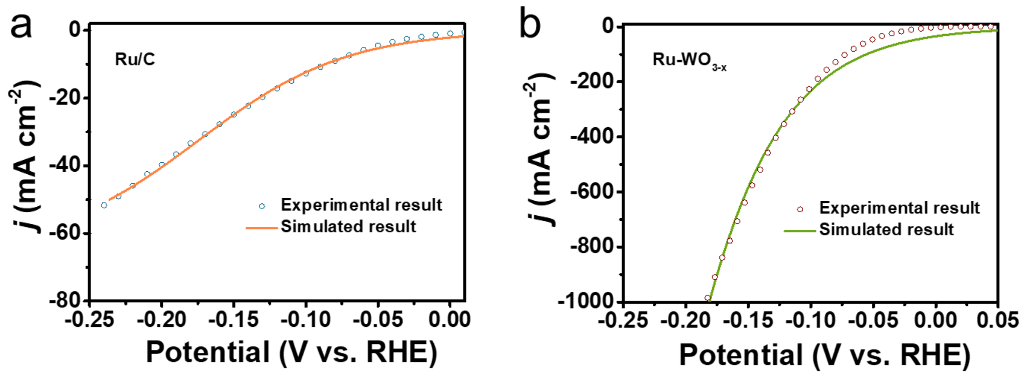

Supplementary Figure 1. Simulated and experimental polarization curves. (a) Comparison of experimental HER polarization curve on Ru/C with the simulated polarization curve. (b) Comparison of experimental HER polarization curve on Ru-WO_{3-x} with the simulated polarization curve.

Supplementary Figure 1 displays the comparison of the experimental HER performance of Ru/C and Ru-WO_{3-x} with the simulated results. It can be observed that the three-step mechanism can well reproduce the experimental data.

Supplementary Table 3. The rate constants of HER on Ru/C and Ru-WO_{3-x} at 40 mA cm^{-2}.

Reaction rate constant	Ru/C ($mol \cdot s^{-1} \cdot m^{-2}$)	Ru-WO_{3-x} ($mol \cdot s^{-1} \cdot m^{-2}$)
K_{101}	3.37×10^{-3}	6.56×10^{4}
K_{201}	1.48×10^{4}	6.41
$K_{201} \cdot \exp\left(\frac{\alpha F\eta}{RT}\right)$	2.37×10^{4}	8.86×10^{-3}
$K_{202} \cdot \exp\left(-\frac{(1-\alpha)F\eta}{RT}\right)$	4.94×10^{2}	2.04×10^{-3}
$K_{301} \times \exp\left(\frac{\alpha F\eta}{RT}\right)$	4.78×10^{4}	1.21×10^{-1}
$K_{302} \cdot \exp\left(-\frac{(1-\alpha)F\eta}{RT}\right)$	4.93×10^{2}	2.03×10^{-3}

Supplementary Table 3 lists the rate constants of HER on Ru/C and Ru-WO_{3-x} at 40 mA cm^{-2}. It is clear to see that the HER rate determining step (RDS) on Ru/C and Ru-WO_{3-x} are obviously different. On Ru/C in neutral pH medium, the HER is rate-limited at the water activation step, while the RDS changes to hydrogen recombination on Ru-WO_{3-x}. As a result, a new microkinetic model without the consideration of water activation, but instead taking hydrogen spillover from WO_{3-x} to Ru into consideration was built to simulate the H coverage on Ru-WO_{3-x} (Supplementary Table 4). θ subscripts $H_{1,ads}$ and $H_{2,ads}$ represent the adsorbed H on WO_{3-x} and Ru, respectively. The simulation results are displayed in Supplementary Figure 2.

Supplementary Table 4. Elementary reaction steps of HER in neutral medium on Ru-WO_{3-x}.

$M_1 + H_2O \underset{K_{102}}{\overset{K_{101}}{\rightleftharpoons}} M_1 \cdot H_{ads} + OH^-$	(13)
$M_1 \cdot H_{ads} + M_2 \underset{K_{202}}{\overset{K_{201}}{\rightleftharpoons}} M_2 \cdot H_{ads} + M_1$	(14)
$M_2 \cdot H_{ads} + H_2O + e^- \underset{K_{302}}{\overset{K_{301}}{\rightleftharpoons}} M_2 + H_2 + OH^-$	(15)
$r_1 = K_{101} \cdot \left(1-\theta_{H_{1,ads}}\right) \cdot \exp\left(\frac{\alpha F \eta}{RT}\right)$	(16)
$r_{-1} = K_{102} \cdot \theta_{H_{1,ads}} \cdot \left(\frac{c_{OH^-}}{c^{\Theta}}\right) \cdot \exp\left(-\frac{(1-\alpha) F \eta}{RT}\right)$	(17)
$r_2 = K_{201} \cdot \theta_{H_{1,ads}} \cdot \left(1-\theta_{H_{2,ads}}\right)$	(18)
$r_{-2} = K_{202} \cdot \theta_{H_{2,ads}} \cdot \left(1-\theta_{H_{1,ads}}\right)$	(19)
$r_3 = K_{301} \cdot \theta_{H_{2,ads}} \cdot \exp\left(\frac{\alpha F \eta}{RT}\right)$	(20)
$r_{-3} = K_{302} \cdot \left(1-\theta_{H_{2,ads}}\right) \cdot \left(\frac{c_{OH^-}}{c^{\Theta}}\right) \cdot \exp\left(-\frac{(1-\alpha) F \eta}{RT}\right)$	(21)

Supplementary Table 5. Reaction rate equations for HER in neutral medium on Ru-WO_{3-x}.

$C_{H_{1,ads}} \frac{d\theta_{H_{1,ads}}}{dt} = r_1 - r_{-1} - r_2 + r_{-2}$	(22)
$C_{H_{2,ads}} \frac{d\theta_{H_{2,ads}}}{dt} = r_2 - r_{-2} - r_3 + r_{-3}$	(23)
$C_{dl} \frac{dE}{dt} = j(t) - \left[\left(r_1 - r_{-1}\right) + \left(r_3 - r_{-3}\right)\right] \cdot F/n$	(24)

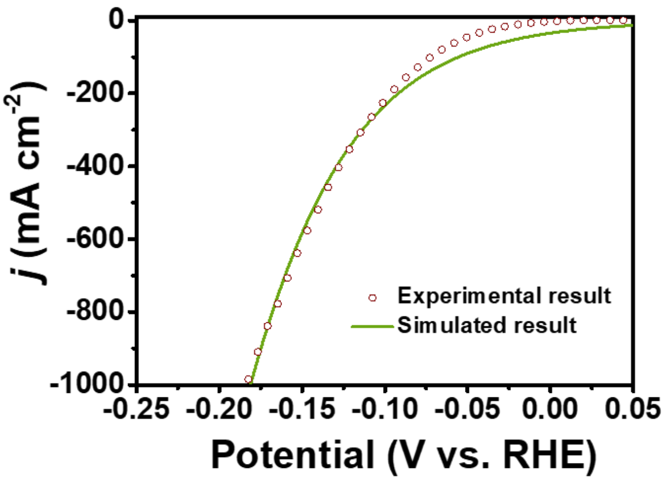

Supplementary Figure 2. Simulated and experimental polarization curves. Comparison of experimental HER polarization curve on Ru-WO_{3-x} with the simulated polarization curve.

Supplementary Table 6. The rate constants of HER on Ru-WO_{3-x} at 40 mA cm^{-2}.

**Reaction rate constant**	**Ru-WO_{3-x} ($mol \cdot s^{-1} \cdot m^{-2}$)**
$K_{201} \cdot \exp\left(\frac{\alpha F \eta}{RT}\right)$	1.46×10^{-1}
$K_{202} \cdot \exp\left(-\frac{(1-\alpha) F \eta}{RT}\right)$	4.34
K_{201}	3.67×10^{-1}
K_{202}	2.48×10^{-2}
$K_{301} \cdot \exp\left(\frac{\alpha F \eta}{RT}\right)$	2.09×10^{-3}
$K_{302} \cdot \exp\left(-\frac{(1-\alpha) F \eta}{RT}\right)$	1.29×10^{1}

Supplementary Note 2. The deuterium isotopic substitution experiment.

Fig. 4b shows the *in-situ* Raman results on Ru-WO_{3-x}/CP recorded in 1.0 M PBS (prepared using D_2O) saturated with N_2. It could be observed that the peak at around 878 cm^{-1} shifted to around 611 cm^{-1}. For Ru-H vibration, the corresponding Raman frequency downward shift ratio (γ) in the isotopic experiment can be analyzed by: $\gamma = \nu(RuD)/\nu(RuH) = \frac{\sqrt{m(Ru)+m(D)}}{\sqrt{m(Ru)\times m(D)}} \div \frac{\sqrt{m(Ru)+m(H)}}{\sqrt{m(Ru)\times m(H)}} = \frac{\sqrt{101.07+2}}{\sqrt{101.07\times 2}} \div \frac{\sqrt{101.07+1}}{\sqrt{101.07\times 1}} = 71.06\%$. As a consequence, the Raman peak at around 878 cm^{-1} should shift to 878 × 71.06% = 623.9 cm^{-1} in the isotopic experiment. Under the actual experimental condition, water solvent effect should be considered and thus the Ru-H vibration can be influenced by the interfacial water molecules, which may exist as RuH-H_2O state through forming hydrogen bond. As a result, in comparison with the calculated result, the Raman frequency of Ru-H can be shifted more obviously in the actual isotopic experiment.

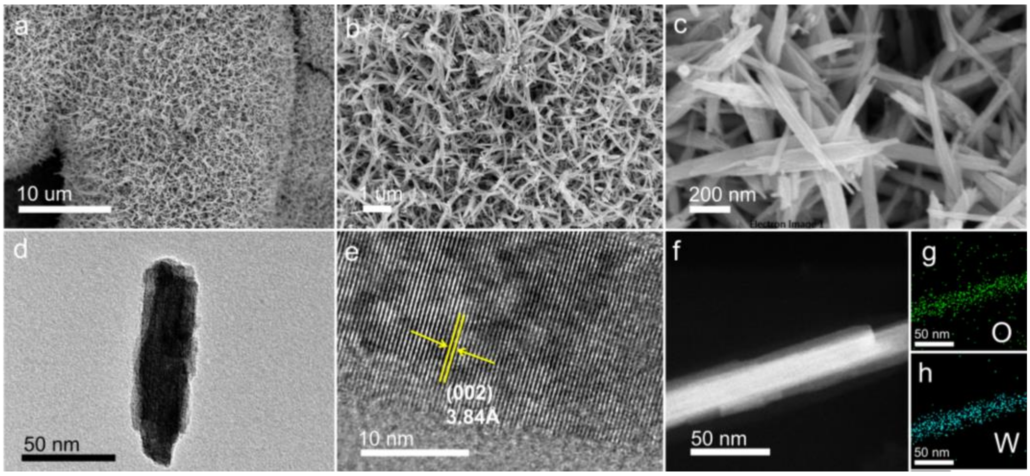

Supplementary Figure 3. Morphological characterization of WO_3/CP. (a-c) SEM images. (d) TEM image. (e) HRTEM image. (f-h) HAADF-STEM image and the corresponding EDX elemental mappings.

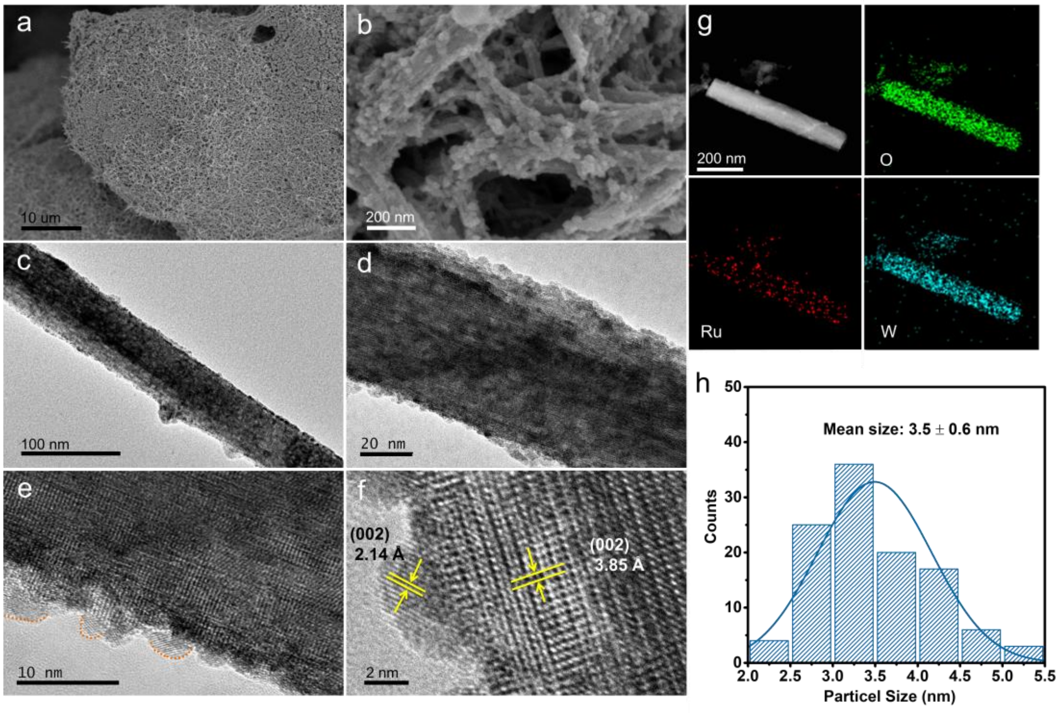

Supplementary Figure 4. Morphological characterization of Ru-WO_{3-x}/CP. (a & b) SEM images. (c-e) TEM images. (f) HRTEM image. (g) HAADF-STEM image and the corresponding EDX elemental mappings. (h) Size distribution profiles of Ru NPS on Ru-WO_{3-x}/CP.

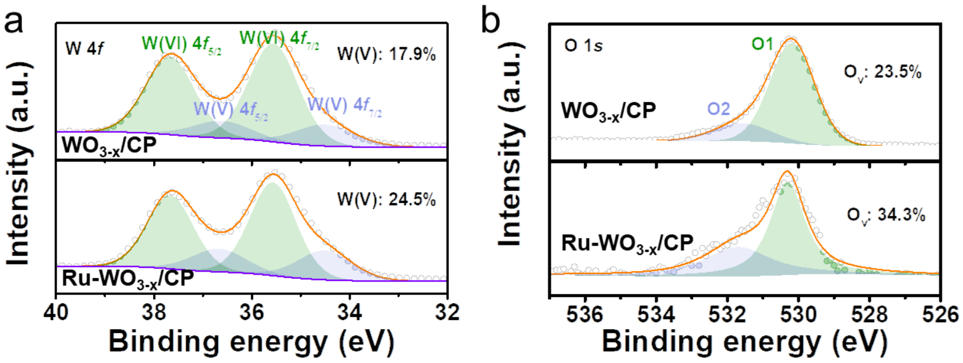

Supplementary Figure 5. XPS spectra of WO_{3-x}/CP and Ru-WO_{3-x}/CP. (a) W 4*f* spectra. (b) O 1*s* spectra.

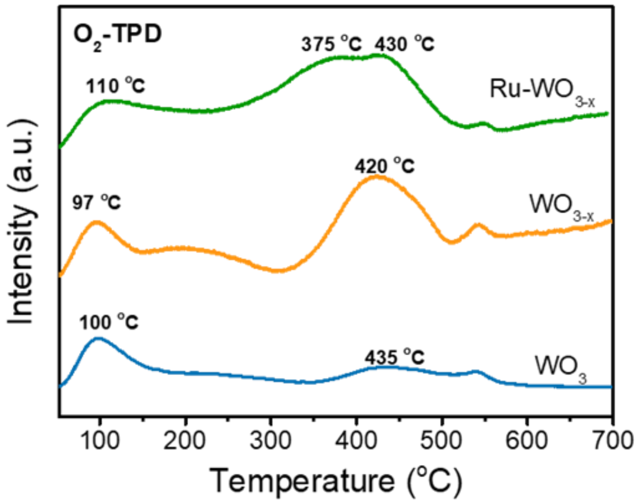

Supplementary Figure 6. O_2-TPD profiles of Ru-WO_{3-x}, WO_{3-x} and WO_3.

To further investigate oxygen vacancies in Ru-WO_{3-x}, we conducted O_2-temperature programmed desorption (O_2-TPD). Generally, the desorption peak at low temperature (< 200°C) is associated to desorption of physically adsorbed oxygen species. The desorption peaks at 200-600 °C correspond to desorption of chemisorbed oxygen species, and the peak above 700 °C is attributed to the release of lattice oxygen[3, 4]. As shown in Figure R3, compared with WO_3, Ru-WO_{3-x} and WO_{3-x} present shifts of desorption peaks of chemisorbed oxygen species to lower temperatures with much higher intensities of desorption peaks at around 400 °C, which should be related to the chemically adsorbed oxygen adjacent to oxygen vacancies. This result reveals oxygen vacancies in WO_{3-x} and Ru-WO_{3-x}, which matches well with the EPR and O 1s XPS results.

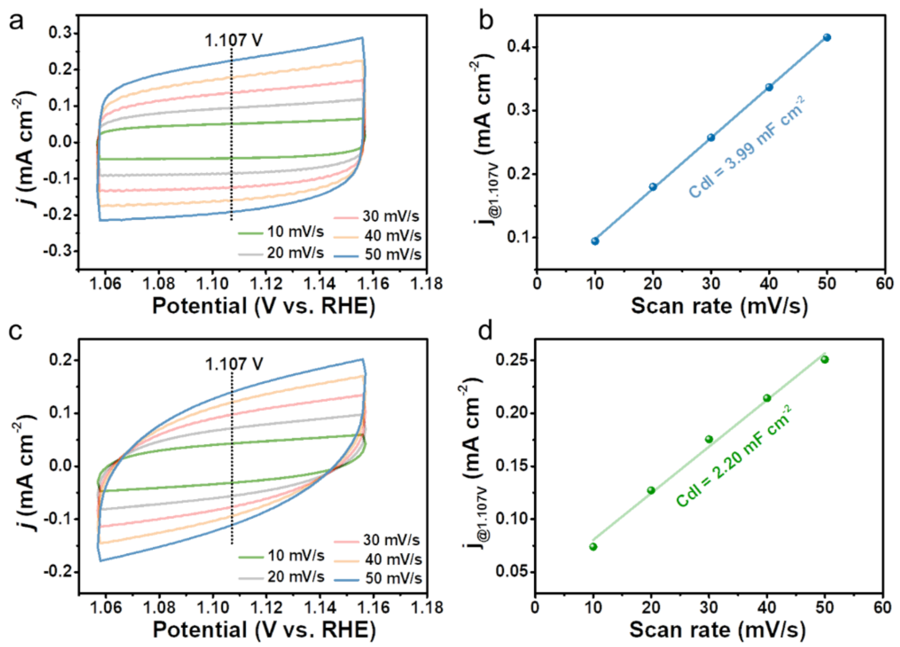

Supplementary Figure 7. CV curves and capacitive currents plotted as the function of scan rates for the calculation of double-layer capacitance. (a) CV curves of Ru/C (5 wt.%) recorded at various scan rates. (b) The capacitive current measured at 1.107 V (vs. RHE) plotted as a function of scan rate of Ru/C (5 wt.%). (c) CV curves of Ru-WO_{3-x} recorded at various scan rates. (d) The capacitive current measured at 1.107 V (vs. RHE) plotted as a function of scan rate of Ru-WO_{3-x}.

Firstly, we calculated the roughness factor (R_f) based on the double layer capacitance (C_{dl}) by the following equation:

$$Rf = \frac{C_{dl}}{0.04}$$

where the C_{dl} (F/g) is the double-layer capacitance of catalysts (Figure R1), and 0.04 (mF/cm^2) is the general double-layer capacitance for a smooth surface for metal oxides[5]. The R_f of Ru/C (5 wt.%) and Ru-WO_{3-x} is calculated to be 100 and 55, respectively. The R_f of Ru-WO_{3-x} is only 55% of Ru/C, indicating that roughness factor should not be a decisive factor in significantly increasing HER activity.

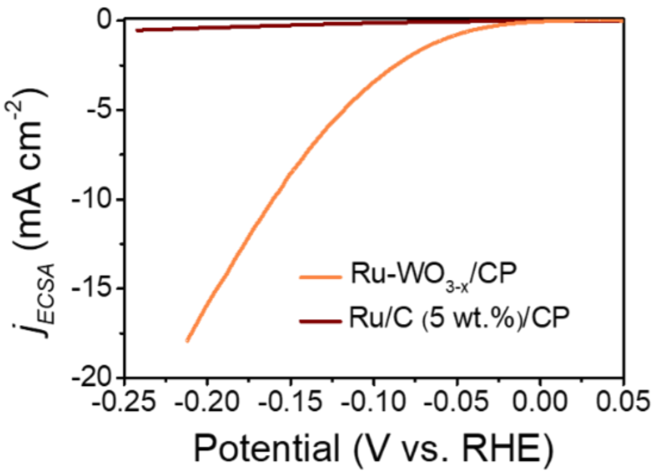

Supplementary Figure 8. LSV curves normalized by ECSA of Ru-WO_{3-x}/CP and Ru/C (5 wt.%)/CP.

Then, we calculated the electrochemically active surface area (ECSA) based on the R_f and obtained the LSV curves normalized by the ECSA. As displayed in Figure R2, Ru-WO_{3-x}/CP still shows much better HER activity than Ru/C (5 wt.%)/CP and the current density of Ru-WO_{3-x}/CP is enhanced by a factor of 34 as compared to the Ru/C (5 wt.%)/CP at the potential of -0.150 V vs. RHE.

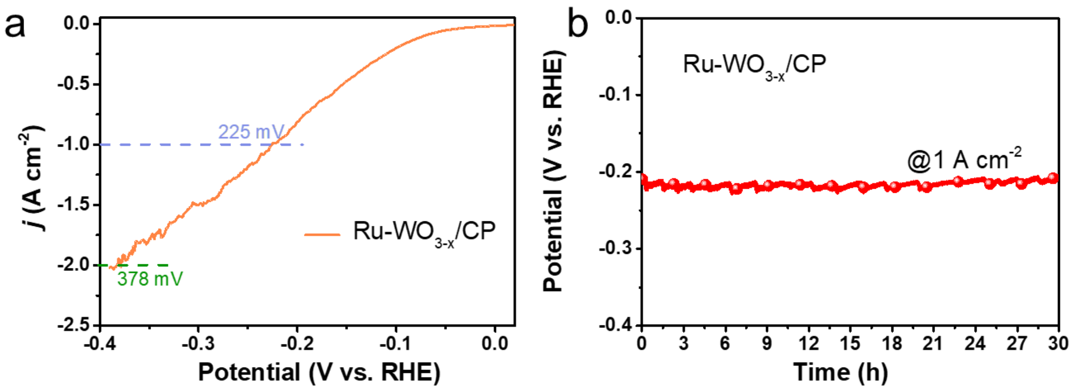

Supplementary Figure 9. HER activity and stability test of Ru-WO3-x/CP in 1.0 M PBS. (a) LSV curve of Ru-WO_{3-x}/CP recorded in 1.0 M PBS. (b) Chronopotentiometric stability of Ru-WO_{3-x}/CP under constant current density of 1 A cm^{-2} in 1.0 M PBS.

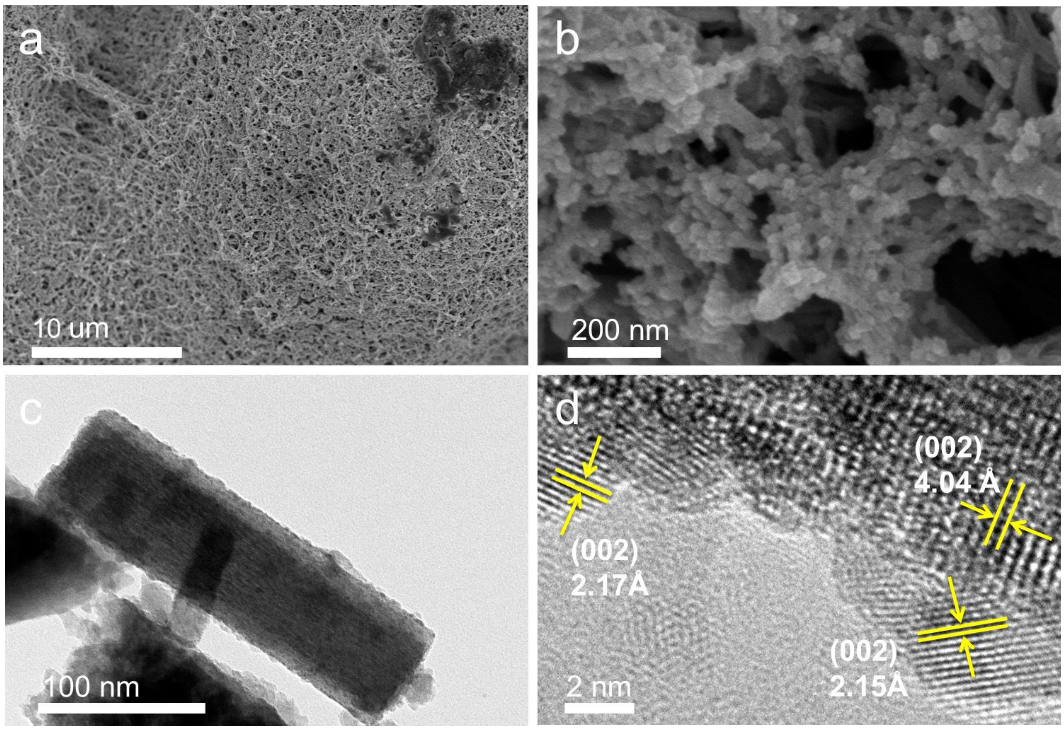

Supplementary Figure 10. Morphological characterization of Ru-WO_{3-x}/CP after stability test. (a & b) SEM images. (c) TEM image. (d) HRTEM image.

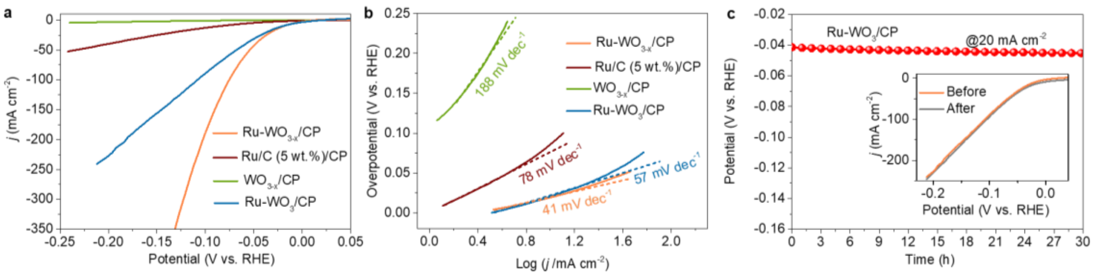

Supplementary Figure 11. HER activity of Ru-WO_3/CP and other catalysts in 1.0 M PBS. (a) LSV curves in 1.0 M PBS. (b) The corresponding Tafel plots. (c) Chronopotentiometric curve recorded at a constant cathodic current density of 20 mA cm^{-2}. Inset compares the LSV curve before and after the stability test.

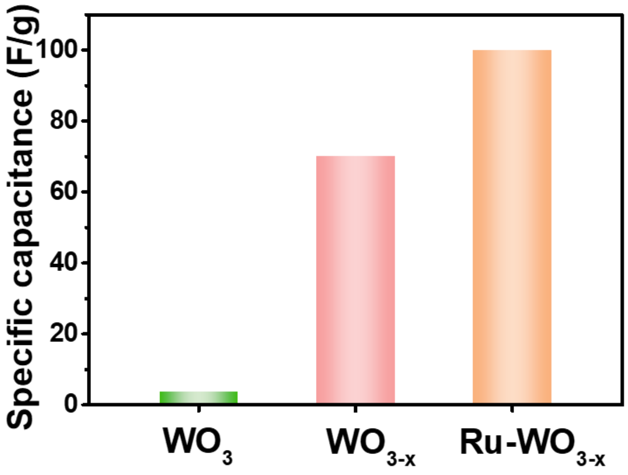

Supplementary Figure 12. Comparison of the capacity for storing protons by specific capacitance. Specific Capacitance of WO_3, WO_{3-x} and Ru-WO_{3-x}.

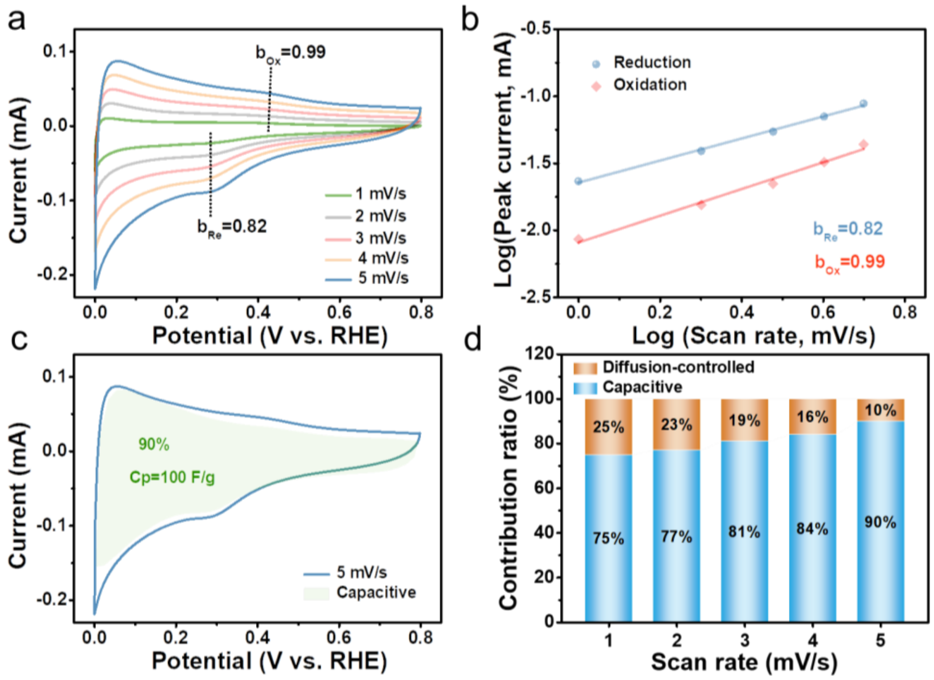

Supplementary Figure 13. Capacitance analysis of Ru-WO_{3-x}. (a) CV curves recorded at various scan rates with calculated b values. Scan rates vary from 1 to 5 mV/s. (b) Analysis of b value for reduction and oxidation peaks. (c) Capacitive contribution at 5 mV/s. (d) Capacitive contribution at various scan rates.

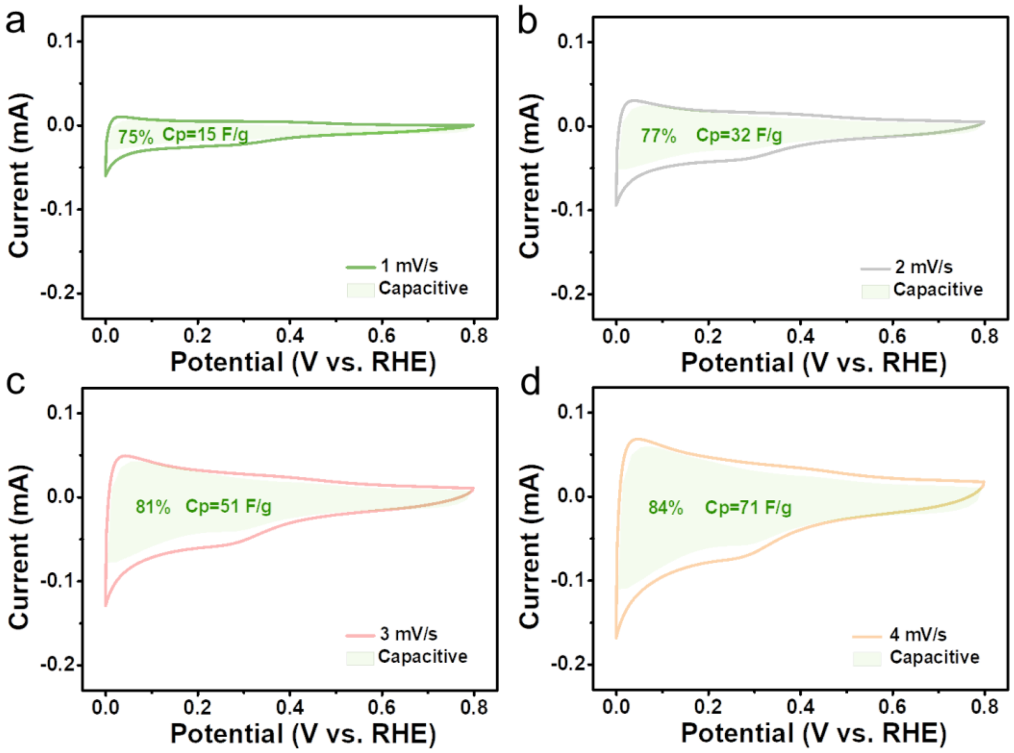

Supplementary Figure 14. Capacitive contribution of Ru-WO_{3-x} at various scan rates.

(a) 1 mV/s. (b) 2 mV/s. (c) 3 mV/s. (d) 4 mV/s.

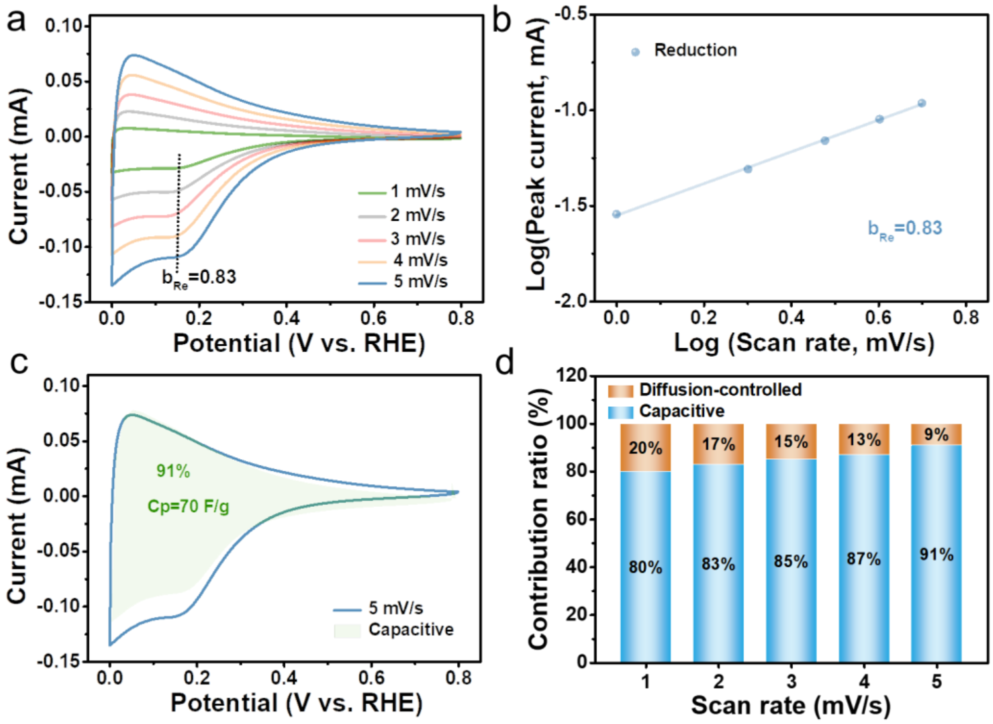

Supplementary Figure 15. Capacitance analysis of WO_{3-x}. (a) CV curves recorded at various scan rates with calculated b values. Scan rates vary from 1 to 5 mV/s. (b) Analysis of b value for reduction peak. (c) Capacitive contribution at 5 mV/s. (d) Capacitive contribution at various scan rates.

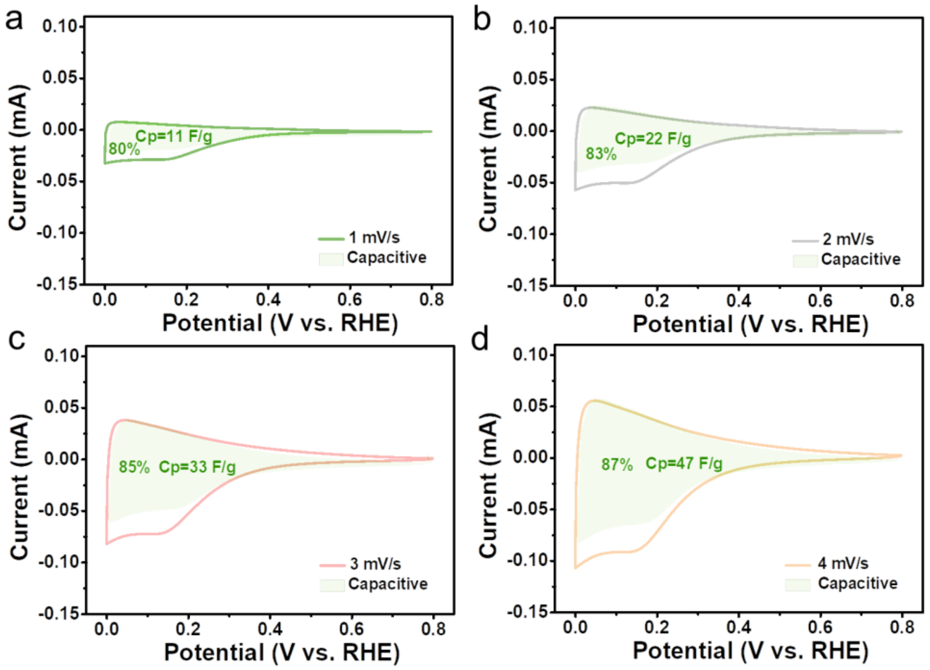

Supplementary Figure 16. Capacitive contribution of WO_{3-x} at various scan rates. (a) 1 mV/s. (b) 2 mV/s. (c) 3 mV/s. (d) 4 mV/s.

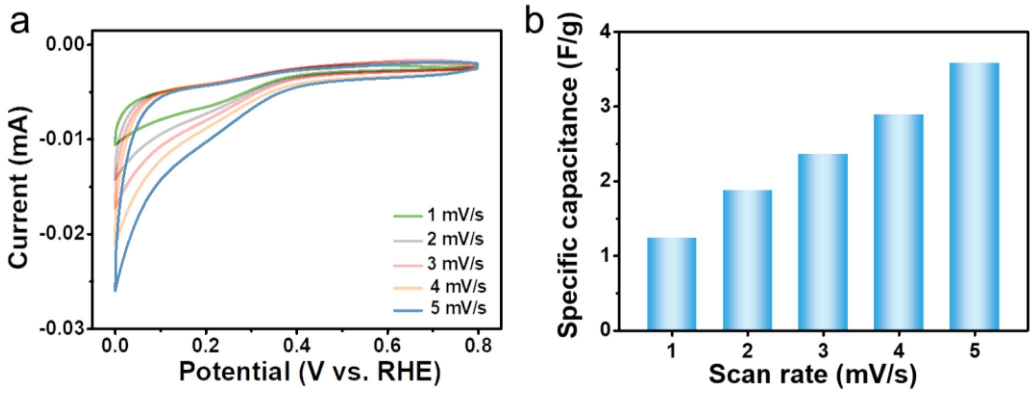

Supplementary Figure 17. Capacitance analysis of WO_3. (a) CV curves recorded at various scan rates. Scan rates vary from 1 to 5 mV/s. (b) Specific capacitance at various scan rates.

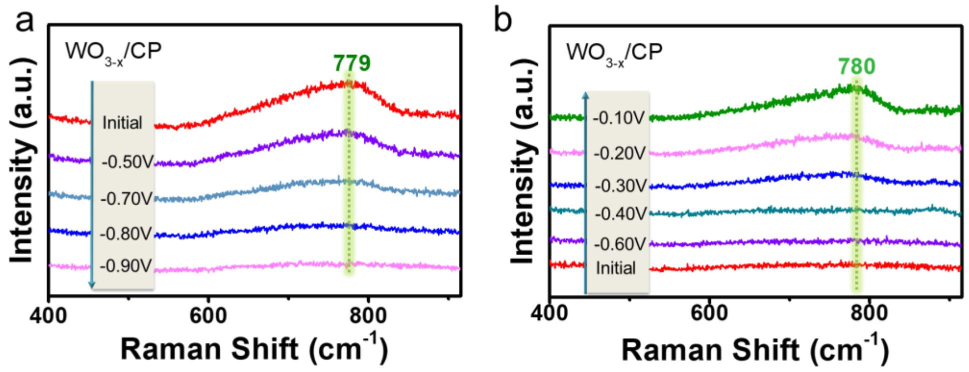

Supplementary Figure 18. *In-situ* Raman analysis of WO_{3-x}/CP in deuterium isotopic substitution experiment. *In-situ* Raman spectra of WO_{3-x}/CP recorded in 1.0 M PBS from -0.5 to -0.9 V vs. Ag/AgCl (a) and sweep back from -0.6 to -0.1 V vs. Ag/AgCl (b).

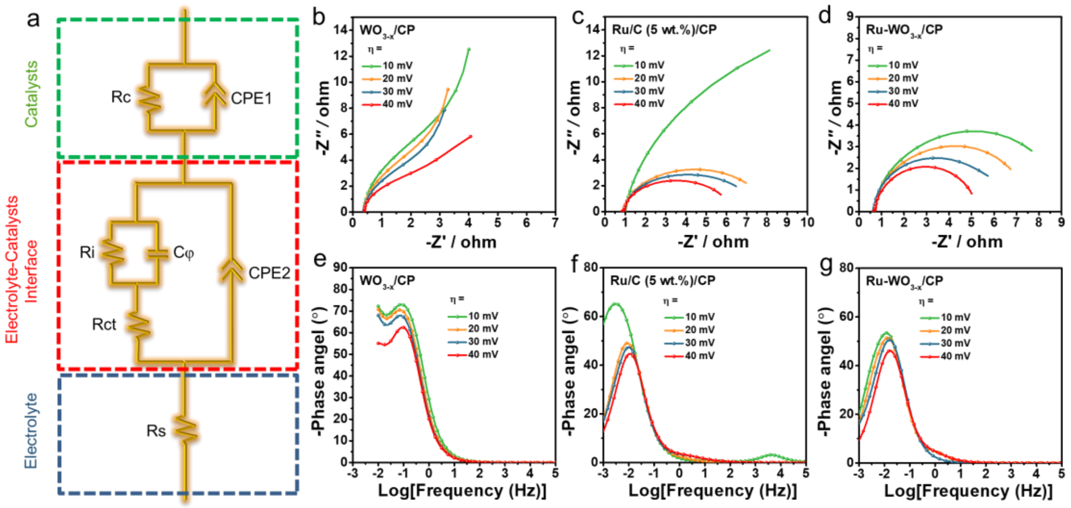

Supplementary Figure 19. Nyquist plots and the corresponding Bode plots at various overpotentials. (a) Schematic diagram of the electrode structure and the equivalent circuit model. (b-d) Nyquist plots of (b) WO_{3-x}/CP, (c) Ru/C (5 wt.%)/CP and (d) Ru-WO_{3-x}/CP. (e-f) Bode plots of (e) WO_{3-x}/CP, (f) Ru/C (5 wt.%)/CP and (g) Ru-WO_{3-x}/CP.

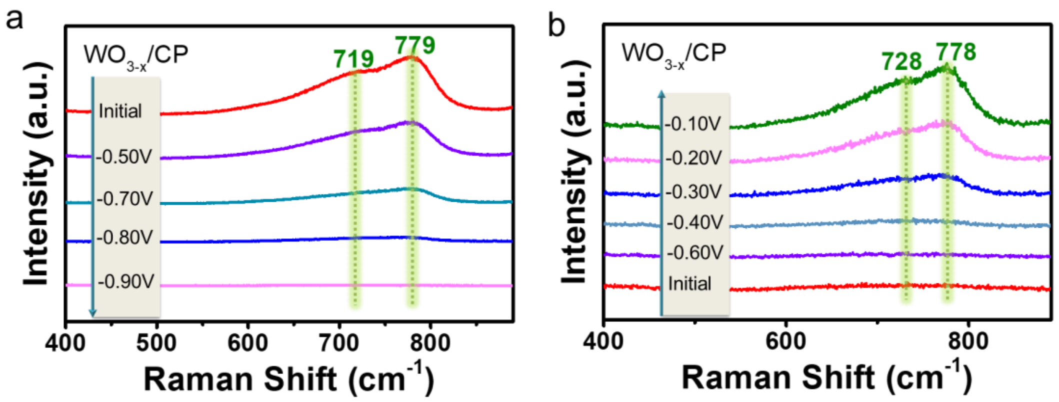

Supplementary Figure 20. *In-situ* Raman analysis of WO_{3-x}/CP. *In-situ* Raman spectra of WO_{3-x}/CP recorded in 1.0 M PBS from -0.5 to -0.9 V vs. Ag/AgCl (**a**) and sweep back from -0.6 to -0.1 V vs. Ag/AgCl (**b**).

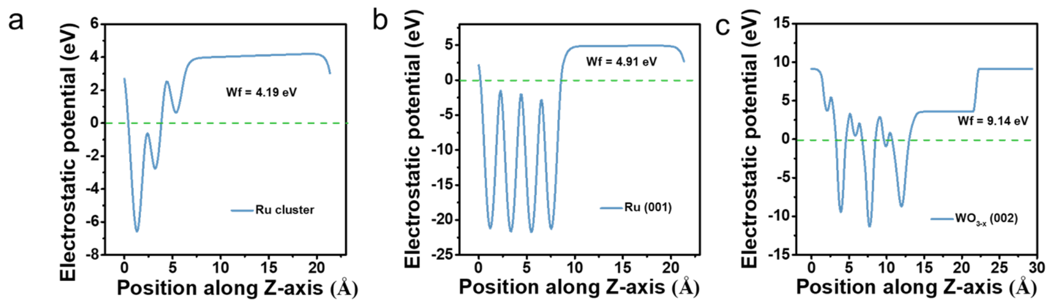

Supplementary Figure 21. Work function estimation. The work function estimated for (a) Ru cluster, and (b) Ru (001) (c) WO_{3-x} (002).

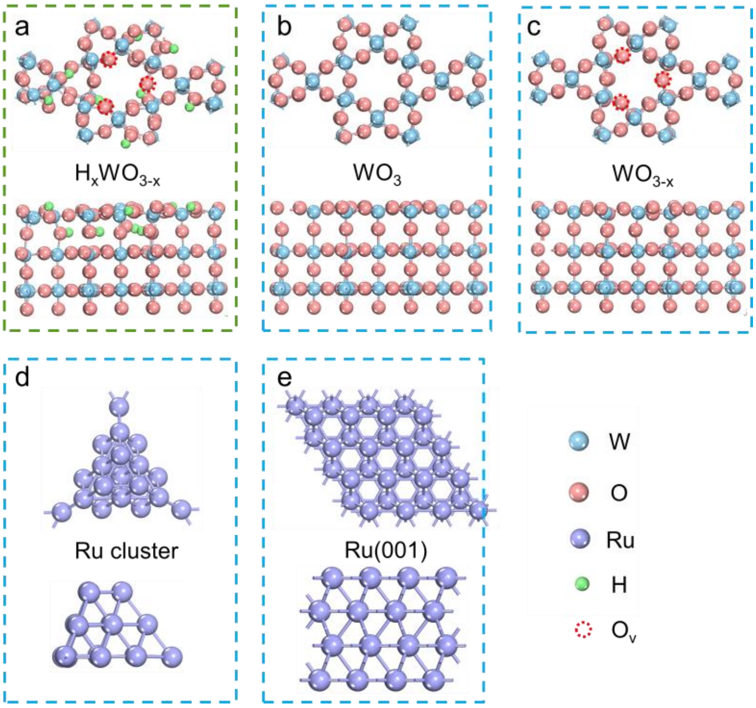

Supplementary Figure 22. Side and top view illustrations of DFT models used for the work function calculation. (a) H_xWO_{3-x} (002). (b) WO_3 (002). (c) WO_{3-x} (002). (d) Ru cluster. (e) Ru (001).

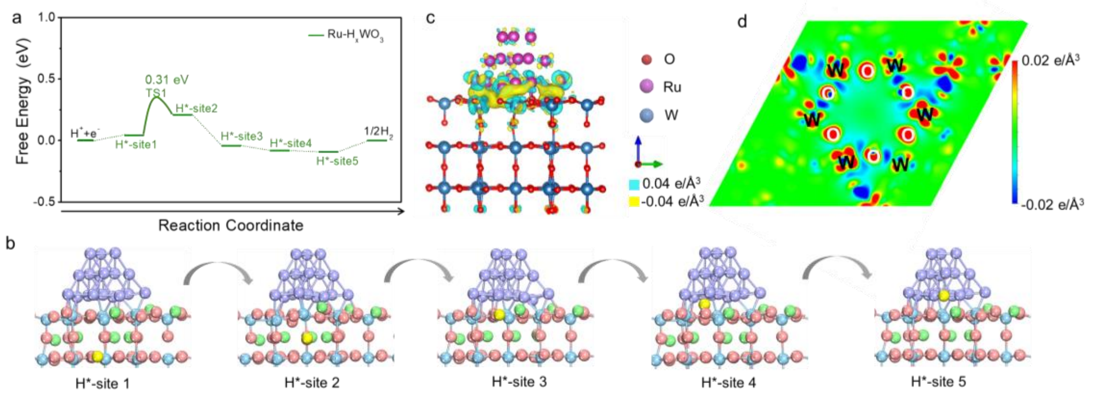

Supplementary Figure 23. DFT calculations of Ru-H_xWO_3. (a) Calculated free energy diagram for HER on Ru-H_xWO_3. (b) The optimized H* adsorption structure on various sites of Ru-H_xWO_3. (c) Electron density difference plot across the Ru-WO_{3-x} interface. Electron accumulation and depletion are indicated in yellow and blue, respectively. (d) The differential charge density maps of Ru-WO_3. From aquamarine to orange indicates the transition from electron depletion to accumulation.

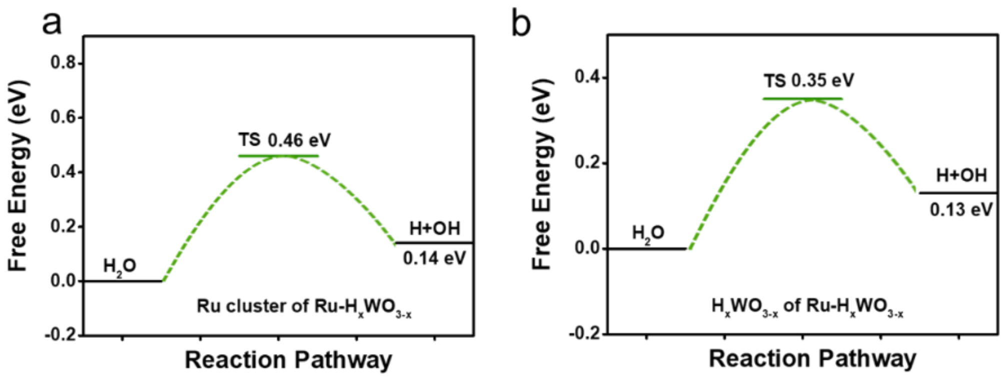

Supplementary Figure 24. Energy barrier of water dissociation process on Ru of Ru-H_xWO_{3-x} (a) and WO_{3-x} of Ru- H_xWO_{3-x} (b). (TS represent the transition states).

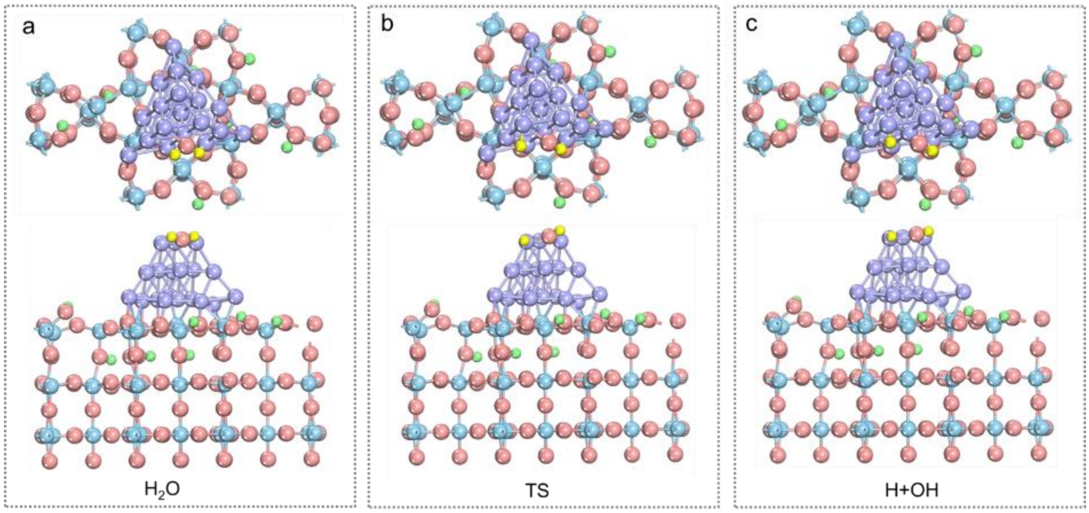

Supplementary Figure 25. Side and top view illustrations of DFT models used for calculating the energy barrier of water dissociation process on Ru of Ru-H_xWO_{3-x}. (a) Initial state. (b) Transition state. (c) Final state. The blue, red and purple balls represent W, O and Ru atoms. The green and yellow balls represent the H atoms inserted into WO_{3-x} and the H atoms involved in HER.

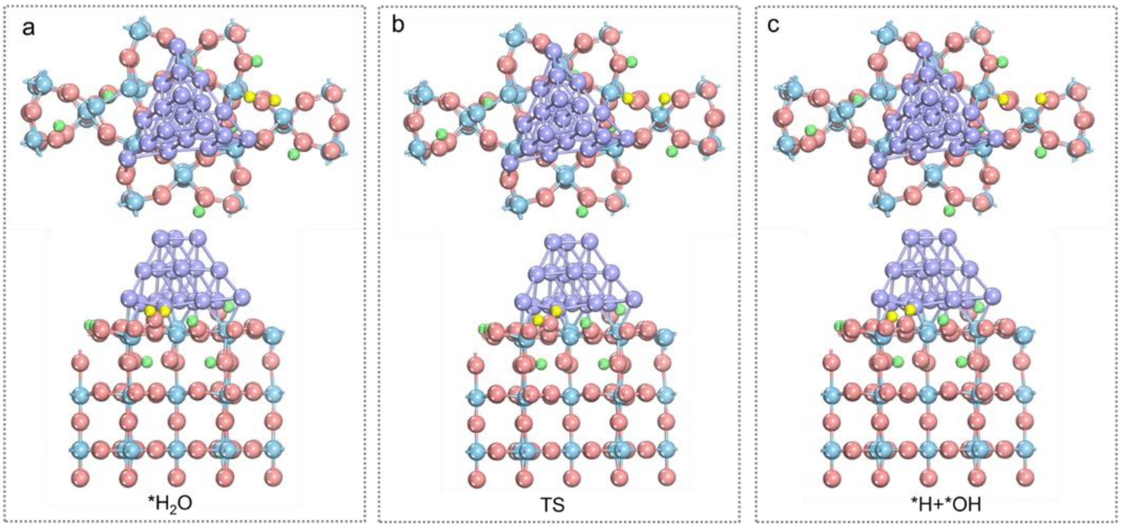

Supplementary Figure 26. Side and top view illustrations of DFT models used for calculating the energy barrier of water dissociation process on WO_{3-x} of Ru-H_xWO_{3-x}. (a) Initial state. (b) Transition state. (c) Final state. The blue, red and purple balls represent W, O and Ru atoms. The green and yellow balls represent the H atoms inserted into WO_{3-x} and the H atoms involved in HER.

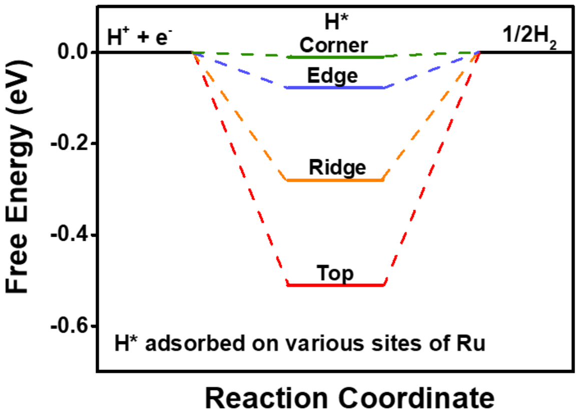

Supplementary Figure 27. Comparison of the hydrogen adsorption on various active sites. Free energy of hydrogen adsorption on Ru-H_xWO_{3-x}.

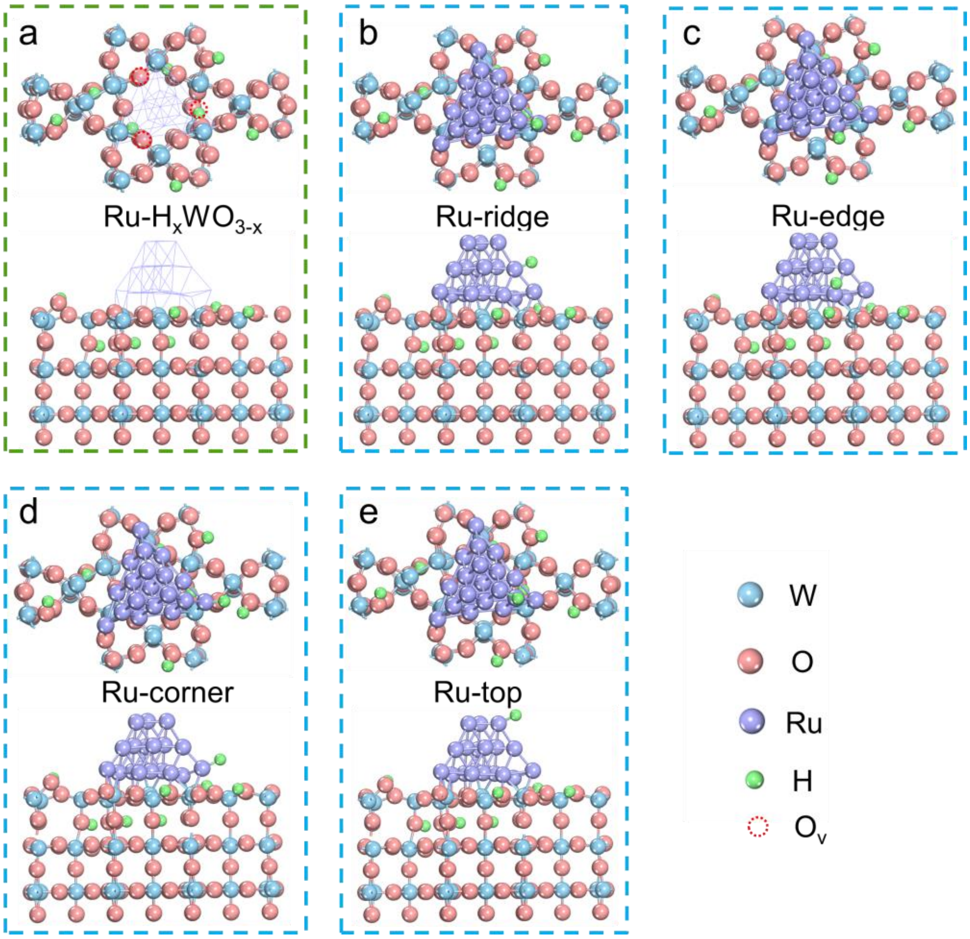

Supplementary Figure 28. Top and side views of DFT models used for calculating the hydrogen adsorption energy of Ru-H_xWO_3. (a) Ru-H_xWO_3. (b) Ridge site of Ru on Ru-H_xWO_3. (c) Edge site of Ru on Ru-H_xWO_3. (d) Corner site of Ru on Ru-H_xWO_3. (e) Top site of Ru on Ru-H_xWO_3.

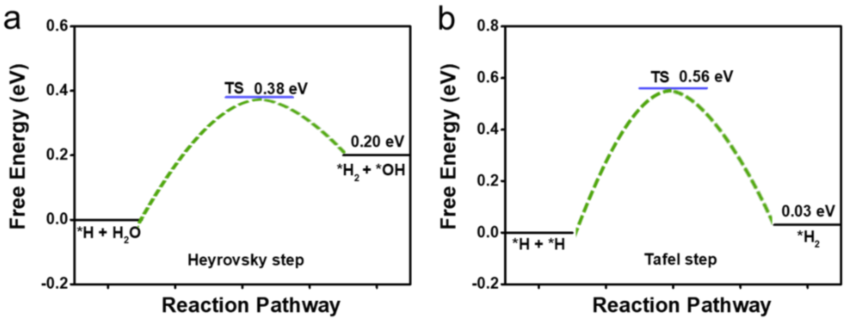

Supplementary Figure 29. Energy barrier of Heyrovsky step (a) and Tafel step (b) on Ru of Ru-H_xWO_{3-x}. (TS represent the transition states).

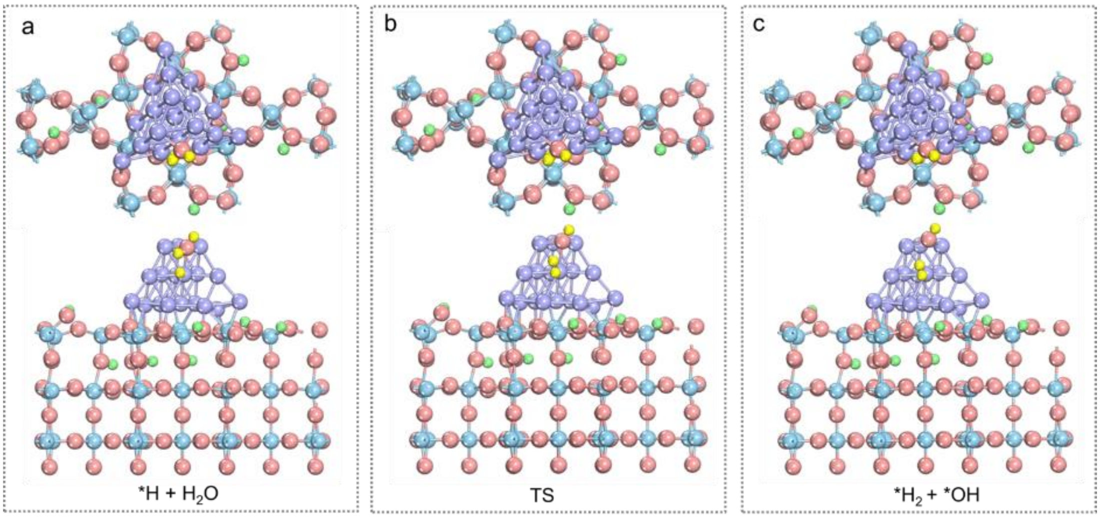

Supplementary Figure 30. Side and top view illustrations of DFT models used for calculating the energy barrier of Heyrovsky step on Ru clusters of Ru-H_xWO_{3-x}. (a) Initial state. (b) Transition state. (c) Final state. The blue, red and purple balls represent W, O and Ru atoms. The green and yellow balls represent the H atoms inserted into WO_{3-x} and the H atoms involved in HER.

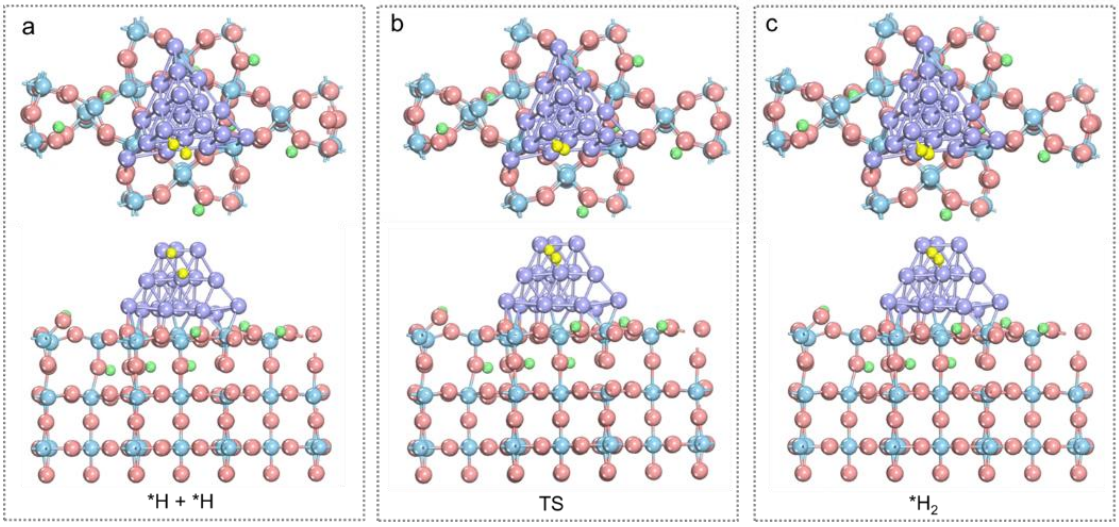

Supplementary Figure 31. Side and top view illustrations of DFT models used for calculating the energy barrier of Tafel step on Ru clusters of Ru-H_xWO_{3-x}. (a) Initial state. (b) Transition state. (c) Final state. The blue, red and purple balls represent W, O and Ru atoms. The green and yellow balls represent the H atoms inserted into WO_{3-x} and the H atoms involved in HER.

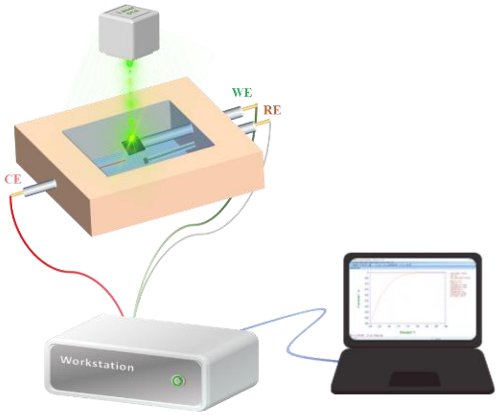

Supplementary Figure 32. Test setup used for Raman in this work. The illustration of in situ Raman spectroscopy setup.

Supplementary Table 7. Ru wt.% determined by inductively coupled plasma optical emission spectrometry (ICP-OES).

**Samples**	Ru wt.%	Ru wt.% after stability test
Ru-WO_{3-x}/CP	5.103	4.981
Ru-WO_3/CP	6.320	6.111
Ru/C(5 wt.%)/CP	4.849	/

Supplementary Table 8. Comparison of HER performance in 1.0 M PBS for Ru-WO_{3-x}/CP and the state-of-the-art HER electrocatalysts.

**Catalyst**	**Electrolyte**	**Tafel slope (mV dec^{-1})**	**Overpotential (mV) @ j=10 mA cm^2**	**Reference**
**Ru-WO_{3-x}/CP**	**1.0 M PBS**	**41**	**19**	**This work**
N–Ni	1.0 M PBS	106	64	Ref.[6]
Pt/np-$Co_{0.85}Se$	1.0 M PBS	35	55	Ref.[7]
PtSA-NT-NF	1.0 M PBS	30	24	Ref.[8]
np-$Co_9S_4P_4$	1.0 M PBS	51	87	Ref.[9]
CoP/Co-MOF	1.0 M PBS	63	49	Ref.[10]
MoP700	1.0 M PBS	79	196	Ref.[11]
$Ni_{0.1}Co_{0.9}P$	1.0 M PBS	103	125	Ref.[12]
$NiCo_2P_x$	1.0 M PBS	63.3	63	Ref.[13]
$Ni_{0.89}Co_{0.11}Se_2$	1.0 M PBS	78	82	Ref.[14]

Mn-Co-P/Ti	1.0 M PBS	82	86	Ref.[15]
$Co_{0.6}Fe_{0.4}P$/CNT	1.0 M PBS	80	105	Ref.[16]
$Cu_{0.08}Co_{0.92}P$ NAs/CP	1.0 M PBS	83.5	81	Ref.[17]
Fe–O–P NRs/CC	1.0 M PBS	47	96	Ref.[18]
MoP NA/CC	1.0 M PBS	94	187	Ref.[19]
N-Co_2P/CC	1.0 M PBS	68	42	Ref.[20]
Karst NF	1.0 M PBS	99	110	Ref.[21]
CoS_x-(0.2-0.02)-12	1.0 M PBS	76	168	Ref.[22]
SiO_2/PPy NTs–CFs	1.0 M PBS	100.2	192	Ref.[23]
Hollow $Ni(S_{0.5}Se_{0.5})_2$	1.0 M PBS	81	124	Ref.[24]
MoP/MoS_2	1.0 M PBS	48	96	Ref.[25]
Co-HNP/CC	1.0 M PBS	38	85	Ref.[26]

Supplementary Table 9. Fitted data of EIS Nyquist plots by the equivalent circuit for Ru-WO_{3-x}/CP, WO_{3-x}/CP and Ru/C (5 wt.%)/CP.

Catalyst	η(mV)	R_s	CPE_1	R_{ct}	$C_φ$(F)	R_i	CPE_2	R_c
**WO_{3-x}/CP**	10	0.21	0.719	16.83	0.705	17.9	0.009	37.2
	20	0.39	0.931	12.45	0.973	10.84	2.461	35.6
	30	0.39	1.893	10.52	1.680	7.05	2.820	35.1
	40	0.40	1.075	6.132	2.258	1.05	0.007	36.1
**Ru/C(5 wt.%)/CP**	10	0.63	2.059	1.052	2.140	6.454	0.001	6.62
	20	0.66	0.269	0.091	4.422	4.051	9.165	7.02
	30	0.65	1.928	0.083	8.371	1.564	10.98	6.22
	40	0.65	1.990	0.074	10.75	0.283	9.066	7.08
**Ru-WO_{3-x}/CP**	10	0.84	0.004	0. 906	1.660	7.028	6.547	3.648
	20	0.82	0.359	0.506	8.067	5.571	0.002	3.585
	30	0.86	1.629	0.125	24.91	3.337	10.58	3.163
	40	0.83	1.639	0.116	57.98	1.223	7.521	4.029

Supplementary References

1. Subbaraman, R., et al. Enhancing hydrogen evolution activity in water splitting by tailoring Li^+-$Ni(OH)_2$-Pt interfaces. *Science* **334**, 1256-1260 (2011).
2. Wang, F., et al. Into the "secret" double layer: Alkali cation mediates the hydrogen evolution reaction in basic medium. *J. Energy Chem.* **51**, 101-104 (2020).
3. Wang, Z., Wang, W., Zhang, L., Jiang, D. Surface oxygen vacancies on Co_3O_4 mediated catalytic formaldehyde oxidation at room temperature. *Catal. Sci. Technol.* **6**, 3845-3853 (2016).
4. Yu, W., et al. Improving gas sensing performance by oxygen vacancies in sub-stoichiometric WO_{3-x}. *RSC Adv.* **9**, 7723-7728 (2019).
5. Qin, M., et al. Operando deciphering the activity origins for potential-induced reconstructed oxygen-evolving catalysts. *Appl. Catal., B* **316**, 121602 (2022).
6. You, B., et al. Universal surface engineering of transition metals for superior electrocatalytic hydrogen evolution in neutral water. *J. Am. Chem. Soc.* **139**, 12283-12290 (2017).
7. Jiang, K., et al. Single platinum atoms embedded in nanoporous cobalt selenide as electrocatalyst for accelerating hydrogen evolution reaction. *Nat. Commun.* **10**, 1743-1751 (2019).
8. Zhang, L., et al. Potential-cycling synthesis of single platinum atoms for efficient hydrogen evolution in neutral media. *Angew. Chem. Int. Ed.* **56**, 13694-13698 (2017).
9. Tan, Y., et al. Three-dimensional nanoporous $Co_9S_4P_4$ pentlandite as a bifunctional electrocatalyst for overall neutral water splitting. *ACS Appl. Mater. Interfaces* **11**, 3880-3888 (2019).
10. Liu, T., et al. CoP-doped MOF-based electrocatalyst for pH-universal hydrogen evolution reaction. *Angew. Chem. Int. Ed.* **131**, 4727-4732 (2019).
11. Xie, X., et al. Electrocatalytic hydrogen evolution in neutral pH solutions: dual-phase synergy. *ACS Catal.* **9**, 8712-8718 (2019).
12. Wu, R., et al. A janus nickel cobalt phosphide catalyst for high-efficiency neutral-

pH water splitting. *Angew. Chem. Int. Ed.* **130**, 15671-15675 (2018).

13. Zhang, R., et al. Ternary $NiCo_2P_x$ nanowires as pH-universal electrocatalysts for highly efficient hydrogen evolution reaction. *Adv. Mater.* **29**, 1605502 (2017).
14. Liu, B., et al. Nickel-cobalt diselenide 3D mesoporous nanosheet networks supported on Ni foam: an all-pH highly efficient integrated electrocatalyst for hydrogen evolution. *Adv. Mater.* **29**, 1606521 (2017).
15. Liu, T., et al. Mn doping of CoP nanosheets array: an efficient electrocatalyst for hydrogen evolution reaction with enhanced activity at all pH values. *ACS Catal.* **7**, 98-102 (2017).
16. Zhang, X., et al. Iron-doped cobalt monophosphide nanosheet/carbon nanotube hybrids as active and stable electrocatalysts for water splitting. *Adv. Funct. Mater.* **27**, 1606635 (2017).
17. Yan, L., et al. Electronic modulation of cobalt phosphide nanosheet arrays via copper doping for highly efficient neutral-pH overall water splitting. *Appl. Catal., B* **265**, 118555 (2020).
18. Huang, J., et al. FeO_x/FeP hybrid nanorods neutral hydrogen evolution electrocatalysis: insight into interface. *J. Mater. Chem. A* **6**, 9467-9472 (2018).
19. Pu, Z., Wei, S., Chen, Z., Mu, S. Flexible molybdenum phosphide nanosheet array electrodes for hydrogen evolution reaction in a wide pH range. *Appl. Catal., B* **196**, 193-198 (2016).
20. Men, Y., et al. Tailoring the electronic structure of Co_2P by N doping for boosting hydrogen evolution reaction at all pH values. *ACS Catal.* **9**, 3744-3752 (2019).
21. Gao, X., et al. Karst landform-featured monolithic electrode for water electrolysis in neutral media. *Energy Environ. Sci.* **13**, 174-182 (2020).
22. He, W., Ifraemov, R., Raslin, A., Hod, I. Room-temperature electrochemical conversion of metal-organic frameworks into porous amorphous metal sulfides with tailored composition and hydrogen evolution activity. *Adv. Funct. Mater.* **28**, 1707244 (2018).
23. Feng, J. X., et al. Silica-polypyrrole hybrids as high-performance metal-free

electrocatalysts for the hydrogen evolution reaction in neutral media. *Angew. Chem. Int. Ed.* **129**, 8232-8236 (2017).

24. Zeng, L., et al. Neutral-pH overall water splitting catalyzed efficiently by a hollow and porous structured ternary nickel sulfoselenide electrocatalyst. *J. Mater. Chem. A* **7**, 16793-16802 (2019).
25. Wu, A., et al. Effective electrocatalytic hydrogen evolution in neutral medium based on 2D MoP/MoS_2 heterostructure nanosheets. *ACS Appl. Mater. Inter.* **11**, 25986-25995 (2019).
26. Liu, B., Zhang, L., Xiong, W., Ma, M. Cobalt-nanocrystal-assembled hollow nanoparticles for electrocatalytic hydrogen generation from neutral-pH water. *Angew. Chem. Int. Ed.* **128**, 6837-6841 (2016).
